# Relationship between prostate-specific antigen, alkaline phosphatase levels, and time-to-tumor shrinkage: understanding the progression of prostate cancer in a longitudinal study

**DOI:** 10.1186/s12894-024-01522-8

**Published:** 2024-07-02

**Authors:** Madiha Liaqat, Rehan Ahmad Khan, Florian Fischer, Shahid Kamal

**Affiliations:** 1 College of Statistical and Actuarial Sciences (CSAS), University of the Punjab, Lahore, Pakistan; 2 Institute of Public Health, Charité – Universitätsmedizin Berlin, Berlin, Germany

**Keywords:** Prostate cancer, Biomarker, Prostate-specific antigen, Alkaline phosphatase, Joint model

## Abstract

**Background:**

This study delves into the complex interplay among prostate-specific antigen, alkaline phosphatase, and the temporal dynamics of tumor shrinkage in prostate cancer. By investigating the longitudinal trajectories and time-to-prostate cancer tumor shrinkage, we aim to untangle the intricate patterns of these biomarkers. This understanding is pivotal for gaining profound insights into the multifaceted aspects of prostate cancer progression. The joint model approach serves as a comprehensive framework, facilitating the elucidation of intricate interactions among these pivotal elements within the context of prostate cancer .

**Methods:**

A new joint model under a shared parameters strategy is proposed for mixed bivariate longitudinal biomarkers and event time data, for obtaining accurate estimates in the presence of missing covariate data. The primary innovation of our model resides in its effective management of covariates with missing observations. Built upon established frameworks, our joint model extends its capabilities by integrating mixed longitudinal responses and accounting for missingness in covariates, thus confronting this particular challenge. We posit that these enhancements bolster the model’s utility and dependability in real-world contexts characterized by prevalent missing data. The main objective of this research is to provide a model-based approach to get full information from prostate cancer data collected with patients’ baseline characteristics ($Age$, body mass index ($BMI$), $Gleason Score$, $Grade$, and $Drug$) and two longitudinal endogenous covariates ($Platelets$ and $Bilirubin$).

**Results:**

The results reveal a clear association between prostate-specific antigen and alkaline phosphatase biomarkers in the context of time-to-prostate cancer tumor shrinkage. This underscores the interconnected dynamics of these key indicators in gauging disease progression.

**Conclusions:**

The analysis of the prostate cancer dataset, incorporating a joint evaluation of mixed longitudinal prostate-specific antigen and alkaline phosphatase biomarkers alongside tumor status, has provided valuable insights into disease progression. The results demonstrate the effectiveness of the proposed joint model, as evidenced by accurate estimates. The shared variables associated with both longitudinal biomarkers and event times consistently deviate from zero, highlighting the robustness and reliability of the model in capturing the complex dynamics of prostate cancer progression. This approach holds promise for enhancing our understanding and predictive capabilities in the clinical assessment of prostate cancer.

## Background

Longitudinal data from biomarkers are usually collected in cancer studies, with the primary outcome variable that consists of time until the pre-specified event occurs, e.g., time to disease progression, recurrence of cancer, or death. Joint models are common to simultaneously analyze longitudinal and event time outcomes by addressing complex issues of informative dropouts, informative censoring, missingness, and association structure [1, 2]. One perspective is to directly introduce longitudinal measurements into Cox’s model as time-varying covariates. However, the risk of bias increases in this approach if observations are measured with error or if there exists intra-individual variability [3].

Two further types of joint modeling approaches have been employed in previous studies: One approach utilizes the factorization of two outcomes as the product of marginal and conditional distributions, and the second approach utilizes random effects to account for the association between outcomes [4, 5]. When multiple outcomes are collected on individuals, the joint model is extended to allow for the correlation structure between different outcomes. More than one similar longitudinal with more than one event time outcome has been analyzed using the usual joint modeling strategy [6].

Simultaneous analysis of multivariate longitudinal and event time outcomes becomes complex to execute when different types of longitudinal outcomes are included in the study [7, 8]. Random-effects modeling strategy is the most frequently applied joint modeling approach, which is well established to analyze continuous and discrete longitudinal data implying an association structure between repeated measures on individuals [9].

Linear mixed-effects models commonly assume longitudinal processes [10, 11] and the proportional hazards model is specified for an event time process [12]. Joint models of multiple outcome processes are connected with parameters using various associated structures such as time-dependent slopes, interaction and lagged effects, cumulative effects, and random-effects parameterization [13].

The presence of missing data leads to a high complexity in longitudinal data due to different follow-up time points. Deletion of missingness has threats related to loss of information and reduction in precise estimates, which produces misleading inferences. The validity of accommodating incompleteness during data analysis depends upon the missingness mechanism. According to Rubin [14], it is based on missingness-completely-at-random (MCAR), missingness-at-random (MAR), and missingness-not-at-random (MNAR) taxonomy, by making assumptions related to observed and unobserved information. MCAR probability does not depend on unobserved or observed information, MAR probability depends upon observed information but it is independent of unobserved information, while the occurrence of MNAR purely depends upon observed and unobserved information.

A single missing value in the data set is replaced with a calculated mean or median or the last observed value is used to fill space of unobserved value. This technique produces biased estimates in the case of MAR [15]. Handling missing data in statistical methodology with a joint evaluation of longitudinal and event time responses provides substantial effects regarding inferences. Selection, pattern-mixture, and shared-parameter models (SPM) are three well-established joint modeling frameworks that are used to apply different factorizations for the joint distribution of outcomes and missing data processes [16]. The SPM approach mostly applies with conditionally independent assumption between outcomes and missingness based on random- effects [17, 18].

Our work aims to develop a joint model for continuous and binary longitudinal responses and an event time outcome with missing covariates. Shared random effects are used to build a joint model for mixed longitudinal outcomes with an-event time outcome; by assuming the existence of sharing effects between longitudinal mixed models and an-event time model. Longitudinal and event time outcomes are conditionally independent of shared parameters and covariates. The ignorability condition fulfills if missingness in time-dependent covariates relates to observed observations but not to unobserved data. A distributional assumption for incomplete observed covariates is inevitable for likelihood inference, while no such assumption is needed for covariates without missingness [19]. We propose a joint model for prostate-specific antigen ($PSA$) and alkaline phosphatase ($ALP$) biomarkers, and time-to-tumor shrinkage status data collected on prostate cancer patients. The proposed model is based on patients’ baseline characteristics as covariates with time-dependent endogenous covariates.

## Methods

### Notation and definitions

The methodology is based on formulation of a joint model for bivariate longitudinal responses, one continuous and normally distributed and the other one binary. Let ${y}_{1ij}$ and ${y}_{2ij}$ be the continuous and binary components of ${j}^{th}$ outcome at time point ${t}_{ij}$ where $i=\text{1,2},3,\dots .,n.$ Let ${y}_{i}={({y}_{1i}^{\top },{y}_{2i}^{\top })}^{\top }$ be the bivariate longitudinal outcome vector for $i$, such that ${y}_{hi}={({y}_{hi1},{y}_{hi2},{y}_{hi3},\dots .,{y}_{hij})}^{\top }$ where $h=\text{1,2}, j=\text{1,2},\dots .,{n}_{hi}$ is an ${n}_{hi}$ column vector for ${h}^{th}$ longitudinal responses for subject $i$.

For bivariate responses, the generalized linear mixed effects model takes the form as,

1 $$E\left({y}_{hi}|{u}_{hi}\right)={g}_{h}\left({X}_{hi}{\beta }_{h}+{Z}_{hi}{u}_{hi}\right), h=\text{1,2}$$

Where, ${g}_{h}\left(.\right)$ denotes bivariate link functions for mixed data types. ${X}_{hi}$and ${\beta }_{h}$ represent the ${n}_{hi}\times {p}_{h}$ design matrix of covariate values of fixed effects ${p}_{h}$ effects. In the same way, ${Z}_{hi}$ and ${u}_{hi}$ denote a ${n}_{hi}\times {q}_{1}$ design matrix of covariates of ${q}_{h}$ dimensional vector of normally distributed random effects with mean vector $0$ and covariance-matrix $\varSigma$. It is assumed that elements of ${y}_{hi}$ are independent conditional on ${u}_{hi}.$

The generalized linear mixed effects model (1) is written using an identity link for continuous response and a logit link for binary response [20], respectively as,

2 $$E\left({y}_{1i}|{u}_{1i}\right)={X}_{1i}{\beta }_{1}+{Z}_{1i}{u}_{1i},$$

3 $$logit\left(\text{Pr}\left({y}_{2i}=1\right)|{u}_{2i}\right)={X}_{2i}{\beta }_{2}+{Z}_{2i}{u}_{2i}.$$

It is assumed that ${u}_{1i}$ follows a normal distribution with zero mean vector and variance-covariance matrix $\varSigma$, and ${u}_{2i}$ is proportional to ${u}_{1i}$. It is written as ${u}_{2i}={\mathsf{A}}_{0}{u}_{1i}$, where ${\mathsf{A}}_{0}$ denotes a diagonal matrix of unknown constants.

True event time ${T}_{i}$ and censoring time ${C}_{i}$ are defined for individual $i$, using observed event time ${T}_{i}^{*}=\text{m}\text{i}\text{n}({T}_{i},$ Ci). Censoring status is represented with ${ \varDelta }_{i}=I({T}_{i}\le {C}_{i})$, by assuming non-informative censoring criteria [21]. It is assumed that event time for ${i}^{th}$ individual is associated with continuous and binary longitudinal responses via shared random effects ${u}_{1i}$ and ${u}_{2i}$. Specifically, the hazard function for $ith$ individual at time ${T}_{i}$ is given by a proportional model, given as,

4 $${h}_{i}\left(t|{x}_{3i},{U}_{3i}\right)={h}_{0}\left(t\right)\text{e}\text{x}\text{p}({x}_{3i}^{\top }{\beta }_{3}+{U}_{3i}),$$

where ${h}_{0}\left({T}_{i}\right)$ is the baseline hazard function, assumed to be parametric or left unspecified. ${X}_{3i}$is a ${p}_{3}$ dimensional vector of covariates with regression coefficients ${\beta }_{3}$.

Here ${U}_{3i}$, shared parameters are used to associate the random effects of longitudinal outcomes with the random effect of event time outcome.

The shared random effects joint model becomes,

5 $${{U}_{3i}=\varPsi }_{1}^{\top }{u}_{1i}+{\varPsi }_{2}^{\top }{u}_{2i}+{u}_{3i},$$

where, ${\varPsi }_{1}^{\top },{ and \varPsi }_{2}^{\top }$are associated parameters and ${u}_{3i}$ is a normally distributed frailty term. Longitudinal outcome vector ${y}_{i}$ and an-event time outcome *T* are conditionally independent on covariates and random-effects vector $u$. Let’s combine observed data as,

6 $$\left\{{y}_{hi}\left({t}_{ij}\right),X\left({t}_{ij}\right),Z\left({t}_{ij}\right),{T}_{i}^{*},{\varDelta }_{i},i=\text{1,2},3,\dots .,n,j=\text{1,2},3,\dots .,{n}_{hi},h=\text{1,2}\right\}.$$

The joint distribution of observed data based on conditional independence assumption is given for individuals as,

7 $$f\left({y}_{i},{T}_{i}^{*},{\varDelta }_{i}|{u}_{i};{\uptheta }\right)=f\left({y}_{1i}|{u}_{1i};{\beta }_{1},{\sigma }_{\epsilon 1}^{2}\right)\mathsf{x}f(\left({y}_{2i}|{u}_{2i};{\beta }_{2}\right)\mathsf{x}f\left({T}_{i}^{*},{\varDelta }_{i}|{u}_{i};{\beta }_{3},\varPsi ,{\sigma }_{3}^{2}\right),$$

where θ denotes the parameter vector and *f*(・) denotes the probability density function. The log-likelihood for the observed data is expressed by,

8 $$\sum\nolimits_{i=1}^{N}log{\int }_{{u}_{i}}\prod\nolimits_{i=1}^{{n}_{1i}}[\frac{1}{\sqrt{2\pi {\sigma }_{\epsilon }^{2}}}\text{e}\text{x}\text{p}\{-\frac{1}{2{\sigma }_{{\epsilon }_{1}}^{2}}{\left({y}_{1ij}-{x}_{1ij}{\beta }_{1}-{z}_{1ij}{u}_{1i}\right)}^{2}\}]\times \prod\nolimits_{j=1}^{{n}_{2i}}\left[{\left\{\frac{1}{1+\text{exp}\left(-{x}_{2ij}{\beta }_{2}-{z}_{2ij}{u}_{2i}\right)}\right\}}^{{y}_{1ij}}{\left\{\frac{\text{exp}\left(-{x}_{2ij}{\beta }_{2}-{z}_{2ij}{u}_{2i}\right)}{1+\text{exp}\left({-x}_{2ij}{\beta }_{2}-{z}_{2ij}{u}_{2i}\right)}\right\}}^{{1-y}_{2ij}}\right]\times [{h}_{0}\left({T}_{i}^{*}\right)\text{e}\text{x}\text{p}\{{x}_{3i}^{T}{\beta }_{3}+ {U}_{i}\}{]}^{{\varDelta }_{i}}\text{e}\text{x}\text{p}\{-{\int }_{0}^{{T}_{i}^{*}}{h}_{0}\left(s\right)\text{e}\text{x}\text{p}\{{x}_{3i}^{T}{\beta }_{3}+{U}_{i}\}ds\}\times f({u}_{i}\left|\varSigma \right)d{u}_{i}.$$

If associated parameters $\varPsi ={({\varPsi }_{1}^{\top },{ \varPsi }_{2}^{\top })}^{\top }=\left(\text{0,0}\right)$ are zero, there exists no association among the three outcome processes, in this case, joint modeling is not necessary and separate models can be used. In Bayesian inference, Markov-Chain-Monte-Carlo methods including Gibbs sampler and Metropolis-Hastings algorithm implemented to draw samples iteratively from conditional posterior distribution [22]. The conditional posterior distribution is written with joint prior distribution $p\left({\theta }_{{y}_{1i}}^{{\prime }},{\theta }_{{y}_{2i}}^{{\prime }},{\theta }_{{T}_{i}^{*}}^{{\prime }},{\theta }_{{\varDelta }_{i}}^{{\prime }},{\theta }_{{u}_{i}}^{{\prime }}\right)$ of all parameters.

Let ${x}_{i}^{mis}$be a vector of time-dependent missing covariates included in the generalized linear model, which is given as,

9 $$p\left({x}_{ij}|{\theta }_{ij},\tau \right)=\text{exp}\left\{{\alpha }_{i}^{-1}\left(\tau \right)\left(x{\theta }_{ij}-b\left({\theta }_{ij}\right)\right)+c\left(x,\tau \right)\right\}, {x}_{ij} \in {x}_{i}^{mis};$$

the term $\prod _{i\epsilon {x}_{i}^{mis}}{\prod }_{i=1}^{n}p\left({x}_{ij}|{\theta }_{ij},\tau \right)$ be added to the conditional posterior distribution.

Let ${x}_{ij}^{mis }$be missing covariates and ${x}_{ij}^{obs}$ be observed covariates, and $\left\{\left({R}_{ij}, {y}_{ij},{x}_{ij}^{obs},{x}_{ij}^{mis}\right)\right\}$ be full data, where ${R}_{ij}$ is missing data indicator, which takes value $0$ or $1,$ based on observe or missing observations, and $\theta$ is parameter of missingness. ${R}_{ij}$ is independent of missing observations ${x}_{ij}^{mis}$ but may depend on observed values (${y}_{ij},{x}_{ij}^{obs}$). We assume random-effects association for two heterogeneous longitudinal processes, this parameterization is more meaningful for our case, where random-intercept and random-slope are assumed for longitudinal sub-models. Both frequentist and Bayesian statisticians have agreed on the fact of inaccurate estimates in case of not incorporating missing covariates in data analysis [19]. In a frequentist context, many parameters of the likelihood function are not identifiable due to missing covariates, Bayesian analysis provides a solution to this problem by appropriately choosing proper prior distributions, using informative priors. Informative priors add information about unknown parameters when based on true available historical data. If no previous knowledge is available, non-informative prior distribution and Gibbs sampling are used to combine with the Metropolis-Hastings algorithm under full conditional distribution of each model parameter [23]. We implement Bayesian thinking for parameter inferences, using the Gibbs sampling algorithm with the help of ‘OpenBUGS’ [24]. Weak-informative priors are chosen, and fixed effects parameters are assumed to be distributed normally with large variance components. For variances of error terms inverse-gamma prior distributions and for covariances of random-effects inverse-Wishart distributions are applied as priors. The implementation code is available upon request from the corresponding author.

#### Application to mixed longitudinal (PSA, ALP) and a time-to-tumor status prostate cancer study

Prostate cancer being the major cause of cancer deaths in men has increased the disease burden in terms of diagnosis and progression to assess the effectiveness of treatment. In the era from 1988 to the mid-1990s, $PSA$ was the only serum marker for prostate cancer and it is still used for diagnosis, measuring treatment efficacy, and recurrence of prostate cancer [25, 26]. $ALP$ is one of the older biomarkers for investigating and monitoring prostate cancer. Furthermore, it is a reliable indicator of bone metastases [27, 28]. Various authors have proved an increase in the clinical effectiveness of simultaneous measurement of $PSA$ and $ALP$ in prostate cancer patients [29, 30].

Our motivation for joint modeling of longitudinal and event time outcomes arises from a prostate cancer dataset collected from one of the most renowned public hospitals in Pakistan. This dataset includes patients who underwent various treatments and were followed to monitor PSA and ALP measurements until tumor shrinkage or censoring occurred. To study the effects of biomarkers on event time, we jointly modeled longitudinal PSA and ALP biomarkers along with tumor shrinkage outcomes.

The study included patients diagnosed with prostate cancer as their primary disease, who provided blood samples during follow-up visits spaced 28 to 30 days apart. The demographic characteristics of the study sample showed an age distribution ranging from 50 to 85 years, with a mean age of 68 years. Clinically, the patients varied in disease stage, with a significant number in advanced stages (stages III and IV), indicating a high-risk population.

Treatment histories of the patients included a mix of therapeutic approaches: surgery (prostatectomy), radiation therapy, hormone therapy (androgen deprivation therapy), and combinations thereof. This diversity in treatment history reflects the standard care practices for managing advanced prostate cancer and provides a comprehensive view of the patient population under investigation.

To remove variations caused by different scales of data, we standardized all continuous variables. Specifically, each variable was transformed by subtracting its mean and dividing by its standard deviation, resulting in variables with a mean of zero and a standard deviation of one. This standardization ensures that all continuous variables contribute equally to the model and prevents those with larger scales from dominating the analysis.

Additional preprocessing steps included addressing missing data through multiple imputations to mitigate bias from incomplete datasets. Outliers were identified and assessed, with extreme values either transformed or excluded based on their influence on the overall analysis. Categorical variables were encoded using one-hot encoding to convert them into a format suitable for modeling.

## Results

To jointly model three outcomes the model is specified as,

10 $${y}_{1ij}={\eta }_{1i}\left({t}_{ij}\right)+{U}_{1i}\left({t}_{ij}\right)+{\epsilon }_{ij1}.$$

To model longitudinal $log\left(PSA\right)$ a linear-mixed-effects model with random intercept and slope is specified as,

$$\text{l}\text{o}\text{g}\left({PSA}_{ij}\right)={\beta }_{11}+{\beta }_{12}{t}_{ij}+{\beta }_{13}{Age}_{i}+{\beta }_{14}{Platelets}_{i}+{\beta }_{15}{BMI}_{i}+$$

$${\beta }_{16}{Bilirubin}_{i}+{\beta }_{17}{GleasonScore}_{i}+{\beta }_{18}{Grade}_{i}+{\beta }_{19}{Drug}_{i}$$

11 $$+{u}_{10i}+{u}_{11i}{t}_{ij}+{\epsilon }_{1}\left(t\right).$$

The random-intercept ${u}_{10}$ and random-slope ${u}_{11}$ are assumed to follow a bivariate normal distribution with means $0$ and variance-covariance components ${{\sigma }_{u}^{2}}_{10},{{\sigma }_{u}^{2}}_{11},$ and ${\sigma }_{{u}_{10}{u}_{11}}.$ In addition, the random error term ${\epsilon }_{1ij}\sim N(0,{\sigma }_{\epsilon 1}^{2})$ is independent of random effects.

Binary longitudinal outcome ($ALP$) considers a logistic mixed-effects regression model as,

12 $$logit\left(P\left({y}_{2ij}=1\right)\right)= {\eta }_{2i}\left({t}_{ij}\right)+{U}_{2i}\left({t}_{ij}\right),$$

where,

13 $$\begin{aligned} &={\beta }_{21}+{\beta }_{22}{s}_{ij}+{\beta }_{23}{Age}_{i}+{\beta }_{24}{Platelets}_{i}+{\beta }_{25}{BMI}_{i}+\\ &{\beta }_{26}{Bilirubin}_{i}+{\beta }_{27}{GleasonScore}_{i}+{\beta }_{28}{Grade}_{i}+{\beta }_{29}{Drug}_{i}+{{\varPsi }_{1}u}_{10i}+{{\varPsi }_{2}u}_{11i}{t}_{ij}. \end{aligned}$$

The third outcome is time to tumor shrinkage. A Weibull model is proposed such that random- effects are shared among three sub-models,

14 $${T}_{i}\sim {\eta }_{3i}+{U}_{3i},$$

15 $${\eta }_{3i}=\text{e}\text{x}\text{p}({\beta }_{31}+{\beta }_{32}{GleasonScore}_{i}+{\beta }_{33}{Grade}_{i}+{\beta }_{34}{Drug}_{i}+{\varPsi }_{3}{u}_{10i}+{\varPsi }_{4}{u}_{11i}+{u}_{3i}).$$

Considering three sub-models under SPM, random-effects terms are defined as follows:

16 $${U}_{1i}\left({t}_{ij}\right)={u}_{10i}+{u}_{11i}{t}_{ij},$$

17 $${U}_{2i}\left({t}_{ij}\right)={{\varPsi }_{1}u}_{10i}+{\varPsi }_{2}{u}_{11i}{t}_{ij},$$

18 $${U}_{3i}={\varPsi }_{3}{u}_{10i}+{\varPsi }_{4}{u}_{11i}+{u}_{3i},$$

It is to be assumed that $${\epsilon }_{ij}$$∼*N (*0, $${\sigma }_{\epsilon }^{2}$$*)*, $${\epsilon }_{ij{\prime }s}$$ indicates error components. The regression coefficients $${\beta }_{11}, \dots .,{\beta }_{19},{\beta }_{21},\dots .,{\beta }_{29},$$ and $${\beta }_{31},\dots .,{\beta }_{34}$$ are fixed effects unknown parameters. In these sub-models, $${Age}_{i}$$ is the baseline age of patients in years, $${Platelets}_{i}$$ counts are recorded over time, $${BMI}_{i}$$ of patients are recorded at entry-level, and $${Bilirubin}_{i}$$ measurements are taken at each time of measurement.$${GleasonScore}_{i},$$$${Grade}_{i},$$ and $${Drug}_{i}$$are binary covariates.

Estimation and inference of our proposed joint model are based on conditional independence assumptions. For our analysis, the two longitudinal biomarkers constitute the joint model by adding additional variance-covariance parameters of the random effects. It is also assumed that given the observed history, the right censoring and observation time of visits are independent of the true event times and future biomarkers’ measurements.

The parameter estimates (Est.), standard deviation (S.D.), and 95% credible intervals (CI) for the prostate cancer dataset were obtained using the proposed joint model, focusing on longitudinal PSA and ALP biomarkers and time-to-tumor shrinkage outcomes. These results, summarized in Table 1, provide insights into the magnitude and direction of associations between covariates and prostate cancer progression.

**Table 1:** Parameter estimates (Est.), standard deviation (S.D.) and 95% credible interval (CI) of PCa data, by applying our proposed joint model

**Parameter**	**Est.**	**S.D.**	**95% CI**
**log(** ${\varvec{P}}{\varvec{S}}{\varvec{A}}$**) biomarker**			
Intercept	0.900	0.100	0.800, 1.100
Obstime	-7.000	0.100	-7.200, -6.800
Age	0.200	0.000	0.200, 0.300
Platelets	0.200	0.000	0.200, 0.300
Body mass index (BMI)	-0.100	0.000	-0.200, -0.100
Bilirubin	0.100	0.000	0.000, 0.200
Gleason Score	1.900	1.300	0.400, 3.500
Grade	-0.900	1.300	-2.500, 0.800
Drug	0.400	0.200	0.100, 0.700
${\sigma }_{\upvarepsilon}^{2}$	1.100	0.000	1.100, 1.200
${\sigma }_{{u}_{1}}^{2}$	0.400	0.000	0.400, 0.500
${\varvec{A}}{\varvec{L}}{\varvec{P}}$** biomarker**			
Intercept	1.900	0.300	1.300, 2.600
Obstime	1.500	0.800	0.200, 3.200
Age	-0.300	0.100	-0.500, -0.100
Platelets	0.500	0.100	0.300, 0.800
Body mass index (BMI)	-0.300	0.100	-0.500, -0.200
Bilirubin	4.800	0.500	3.900, 5.700
Gleason Score	4.000	1.600	1.600, 7.200
Grade	-2.500	1.700	-5.700, 0.600
Drug	-1.100	0.600	-2.500, 0.200
${\sigma }_{u2}^{2}$	0.600	0.500	0.100, 1.700
${\sigma }_{{u}_{1}{u}_{2}}$	0.000	0.100	-0.200, 0.100
**Time-to-tumor shrinkage**			
Intercept	-19.400	0.400	-20.300, -18.600
Gleason Score	0.200	0.100	0.100, 0.300
Grade	-0.400	0.100	-0.600, -0.100
Drug	0.200	0.100	0.100. 0.400
${\sigma }_{u}^{2}$	0.300	0.200	0.100, 0.700
$r$	3.500	0.100	3.300, 3.600
$tau$	0.900	0.000	0.800, 0.900
${ \Psi }_{1}$	-0.100	0.200	-0.400, 0.200
${ \Psi }_{2}$	-10.100	5.600	-23.600, -3.100
${ \Psi }_{3}$	-0.500	0.400	-1.100, 0.100
${ \Psi }_{4}$	-0.600	0.300	-1.400, -0.100

Combined analysis of this study aimed to identify the association structure among the three models (linear mixed-effects for $\text{l}\text{o}\text{g}\left(PSA\right)$, logistic mixed-effects for $ALP$, and event of interest “shrinkage of tumor”), which is directly assessed from the associated parameters mentioned in Table 1.

The positive intercepts for both $PSA$ (0.900) and $ALP$ (1.900) biomarkers suggest baseline levels before considering other covariates. $PSA$ levels decrease significantly over time (Est. = -7.000), while $ALP$ levels show an increase (Est. = 1.500), reflecting different temporal dynamics. Higher platelet counts are associated with increases in both $PSA$ (Est. = 0.200) and $ALP$ (Est. = 0.500). Elevated bilirubin shows a slight increase in $PSA$ (Est. = 0.100) but a substantial increase in $ALP$ (Est. = 4.800). Higher $Gleason Scores$ significantly increase both $PSA$ (Est. = 1.900) and $ALP$ (Est. = 4.000) levels. $Drug$ treatment is associated with increases in $PSA$ (Est. = 0.400) and decreases in $ALP$ (Est. = -1.100).

The first associated parameter estimated value is -0.100 which suggests a slight negative effect, although not statistically significant, with the 95% CI ranging from − 0.400 to 0.200, indicating uncertainty about the true effect size. The second associated parameter indicates a more substantial effect compared to the first associated parameter with an estimated value of -10.100, which suggests a significant negative impact, with the 95% CI ranging from − 23.600 to -3.100, indicating a wide range of possible effect sizes. The estimated value of the third associated parameter${\varPsi }_{3}$ is -0.500 which suggests a negative effect, but with a wide 95% CI (-1.100, 0.100), which indicates uncertainty about the true effect size and direction. The estimated value of ${\varPsi }_{4}$ is -0.600 which suggests a moderate negative impact, with a relatively narrow 95% CI from − 1.400 to -0.100, providing more confidence in the estimated effect size.

Results indicate that $PSA$ is a good biomarker in the diagnosis of prostate cancer, but after treatment, it shows different trends for different treatments. $PSA$ values point out successful or unsuccessful prostate cancer treatment. An increased level of $ALP$ in prostate cancer may be an indicator of advanced cancer, as it means cancer is spreading to bones and tissues. That situation is alarming and emphasizes physicians to make decisions regarding advanced therapies and sometimes combinations of androgen deprivation therapy with prostatectomy. The association between $PSA$ and $ALP$ emphasizes taking into account these two biomarkers simultaneously to get insights into prostate cancer progression.

## Discussion

We developed a joint model for $PSA$ and $ALP$ biomarkers and a time-to-tumor shrinkage variable and applied this model to analyze the prostate cancer dataset to find any potential association between biomarkers and tumor status accounting for patients’ baseline characteristics and endogenous $Platelets$ and $Bilirubin$covariates. We applied a joint modeling approach using parametric models for the longitudinal biomarkers, and Cox’s proportional hazards model for time-to-tumor shrinkage. We proposed a joint model by employing a Bayesian framework, including posterior estimates, CI, and DIC model selection criteria. Results from the fitting model indicate that $PSA$ and $ALP$ biomarkers jointly contribute significantly to prostate cancer tumor shrinkage. Prior distributions are chosen taking inspiration from Choi et al. [23].

Results depict the existence of associations among the different outcomes, so separate models may provide inconsistent estimates. Efficiency gain by employing a joint model as compared to separate models for mixed types of longitudinal (continuous ($PSA$) and binary ($ALP$)) outcomes, which are strongly associated with each other, means these outcome processes are dependent. We focus on parametric Cox’s model to analyze right-censored event time data by applying Weibull distribution for Cox’s model. Joint numerical analysis of heterogeneous data is very difficult and computationally challenging, complexity arises due to the existence of missingness in time-dependent covariates ($Platelets$ and $Bilirubin$). Researchers usually apply list-wise deletion or imputation techniques to handle missing data, which has shortcomings of not providing complete information. In this regard, we provide a novel contribution by formulating a joint model for mixed types of responses, incorporating missingness in time-dependent covariates. More importantly, we provide insights into the role of association parameters in joint modeling, very little literature is available on this line of investigation.

It is recommended that prostate cancer patients should be assessed for $PSA$ observations during the first three months after treatment, in some cases $PSA$ measurements become undetectable by the first month after prostatectomy. $PSA$ alone is not an adequate biomarker for prostate cancer progression [31], $ALP$ is another good biomarker that is correlated with $PSA$. Correlation of both biomarkers confirmed that prostate cancer patients should observed for $PSA$ and $ALP$ simultaneously after treatment to confirm treatment effectiveness on tumor shrinkage.

Finally, we utilized shared random effects to characterize the relationship between longitudinal biomarkers. A potential shortcoming of the shared random effects joint modeling approach is that the structure of random effects for longitudinal biomarkers is limited. Other joint model formulations are available as an alternative to shared random effects like pattern mixture and, selection modeling approaches, those are employed and results can be compared.

Joint modeling of longitudinal and event time has previously been applied to get insights into different diseases, the novelty of this research is that we employed a joint model for two prostate cancer longitudinal biomarkers ($PSA$ and $ALP$), and time to tumor shrinkage. This research aims to provide insight into prostate cancer progression by monitoring $PSA$ and $ALP$ for multivariate covariates until tumor status reaches a satisfactory level according to physicians’ directives. The joint modeling approach is an improvement over traditional separate longitudinal and event time models. This study illustrates the usefulness of simultaneous modeling for analyzing the prostate cancer dataset, but the approach is also applicable to a wide variety of diseases, where multiple mixed types of longitudinal responses may have an association with event time response.

SPMs assume that random effects follow distributions such as normal, gamma, or t-distribution to capture individual-specific characteristics in longitudinal outcomes. Departures from these assumptions can introduce bias into estimates. Error terms in these models often assume Gaussian or skewed distributions like gamma or log-normal [32]. Accurate modeling of within-individual correlations, such as autoregressive or compound symmetry structures, is essential to avoid bias. Longitudinal outcome relationships with time can be modeled using parametric or non-parametric approaches, affecting trend interpretation and prediction accuracy. While these assumptions simplify modeling and estimation, violations can lead to bias, necessitating sensitivity analyses for robustness. Limitations of these models include requirements for large sample sizes, computational challenges, and potential difficulties in interpretation, with sensitivity analyses playing a crucial role in identifying sources of bias and enhancing understanding.

In the future, this joint model can be extended to incorporate additional biomarkers and clinical variables to provide a more comprehensive understanding of prostate cancer progression and treatment response. In addition, the inclusion of genetic markers or imaging data could enhance the predictive accuracy of the model.

Advanced machine learning techniques can also be employed within the joint modeling framework. Techniques such as deep learning could capture complex, non-linear relationships between biomarkers and clinical outcomes can potentially lead to more accurate and personalized predictions. Additionally, our future research plan is to investigate the impact of different imputation methods for missing data, including multiple imputation and machine learning-based approaches to enhance the robustness of the findings.

## Data Availability

Data is available from the corresponding author upon reasonable request.

## Abbreviations

ALP: Alkaline phosphatase
BMI: Body mass index
CI: Credible interval
MAR: Missingness-at-random
MCAR: Missingness-completely-at-random
MNAR: missingness-not-at-random
PSA: Prostate-specific antigen
S.D.: Standard deviation
SPM: Shared-parameter models
